# A Framework for Evaluating the Use of Surveillance Systems for Short‐Term Influenza Forecasting

**DOI:** 10.1111/irv.70144

**Published:** 2025-07-29

**Authors:** Negin Maroufi, Lucy Telfar Barnard, Qiu Sue Huang, Gillian Dobbie, Nayyereh Aminisani, Steffen Albrecht, Nhung Nghiem, Michael G. Baker

**Affiliations:** ^1^ University of Otago Wellington New Zealand; ^2^ Institute of Environmental Science and Research Wellington New Zealand; ^3^ University of Auckland Auckland New Zealand; ^4^ Australian National University Canberra Australian Capital Territory Australia

**Keywords:** artificial intelligence, influenza, machine learning, short‐term forecasting, surveillance

## Abstract

**Background:**

Public health surveillance systems need to monitor influenza activity and guide measures to mitigate its high impact on morbidity, mortality and healthcare systems. There is an increasing expectation that surveillance data will support the modeling of future short‐term disease scenarios using artificial intelligence (AI) and machine learning (ML). This study examines how influenza surveillance can support AI/ML‐based short‐term forecasting for influenza at the community and hospital levels in a high‐income country setting (Aotearoa/New Zealand).

**Methods:**

This study used a two‐phase approach. The first phase involved a comprehensive review of government reports, official websites, and literature to characterize existing influenza surveillance systems. The second phase evaluated systems against eight key attributes—timeliness, sensitivity, specificity, representativeness, coverage, robustness, completeness, and historical data—using a five‐level ranking system. Attribute selection was informed by experts' knowledge, ML requirements, and established frameworks. Weighted scores for training and short‐term forecasting capabilities were calculated to determine alignment with AI/ML requirements.

**Results:**

The Southern Hemisphere Influenza and Vaccine Effectiveness Research and Surveillance (SHIVERS) community cohort and Severe Acute Respiratory Infection (SARI) hospital surveillance emerged as the most useful systems, achieving the highest scores in both training and short‐term forecasting in community and hospital settings, respectively. The National Minimum Dataset of hospitalizations and mortality datasets demonstrated strong training potential but are limited in short‐term forecasting due to timeliness constraints. Additionally, laboratory‐based surveillance performs a useful role in bridging community and hospital datasets.

**Conclusions:**

A set of key attributes is useful for assessing which influenza surveillance systems are best aligned with AI/ML training and short‐term forecasting requirements. These attributes distinguished systems that are likely to be the most suitable for modeling future short‐term disease scenarios for influenza at the community and hospital levels in New Zealand. Integrating these data sources could enhance influenza forecasts to improve public health responses and intervention planning.

## 1 Introduction

Influenza remains a significant global public health challenge, causing seasonal epidemics and occasional pandemics, which result in considerable morbidity, mortality, and healthcare burden [1]. Surveillance systems can help mitigate its impact by guiding timely and informed interventions [2, 3, 4]. According to the World Health Organization (WHO), influenza surveillance involves the systematic collection, compilation, and analysis of data to monitor influenza activity within defined populations, ranging from national to regional or group‐specific levels [2]. Surveillance systems range from simple, single‐source data collection to complex multisource electronic systems and community‐based approaches [2, 4]. These systems provide insights into when and where influenza occurs, monitoring virus changes, and assessing the disease's impact on illness, hospitalizations and mortality [2, 3, 4].

Aotearoa/New Zealand has had established respiratory disease surveillance since 1989, as part of the WHO Global Influenza Surveillance Network [5, 6, 7]. The country employs multiple surveillance approaches to gathering information across various levels of severity [6, 8]. Effective interpretation of this data supports public health decision making, particularly in identifying trends and forecasting disease spread [2]. Surveillance system evaluations can help ensure their quality, efficiency, and usefulness [7]. Such evaluations focus on reviewing the system's outputs and overall performance in achieving its goals by applying standards and performance measures [9]. Various frameworks exist for evaluating surveillance systems in the field of public health [3, 9, 10, 11, 12, 13, 14, 15, 16]. These frameworks focus on assessing how well systems meet their objectives and are well established and valuable for system performance assessment. However, they were not designed to assess the suitability of surveillance systems for AI and ML applications.

The emergence of artificial intelligence (AI) and machine learning (ML) technologies offers transformative potential for enhancing the efficiency and predictive capabilities of surveillance systems' data [17]. By integrating real‐world data from existing systems, these technologies can improve influenza forecasting by providing insights into the near future and support timely and informed public health decision making [13, 18, 19]. Certain attributes such as timeliness, data accuracy (incorporating sensitivity and specificity), data representativeness, coverage, robustness, completeness, and longitudinal data availability are key to ensuring the utility of these datasets in predictive models [11, 13, 17, 18].

Building on existing surveillance evaluation frameworks, this paper proposes a complementary approach focusing on which attributes matter for AI/ML applications. The study provides a method to identify which surveillance systems are best suited for ML applications and discusses how they might be improved or integrated. It uses New Zealand influenza surveillance systems as a case study, evaluating and comparing their suitability for short‐term forecasting and predictive model development. Study findings have the potential to improve the usefulness of surveillance data for generating likely scenarios for short‐term influenza forecasts. Such scenarios are important for understanding the influenza season's trajectory in the community and its short‐term impact on healthcare systems, particularly hospitals and primary care services. This study emphasizes achieving optimal predictive accuracy, that is, forecasts that closely reflect actual outcomes, while addressing practical considerations such as timeliness and the efficient use of available resources.

## 2 Method

This study evaluated influenza surveillance systems in New Zealand in two phases.

The first phase aimed to produce a comprehensive description of influenza surveillance systems in New Zealand. Sources included governmental reports from the Ministry of Health, Health New Zealand (Te Whatu Ora), and the New Zealand Institute for Public Health and Forensic Science (formerly the Institute of Environmental Science and Research [ESR]), along with official websites for each surveillance system and relevant published academic literature. A detailed web search was conducted to gather information on these systems (Appendix S1). This search collected definitions and characteristics of the existing influenza surveillance systems in New Zealand (Appendix S1).

The second phase aimed to evaluate the suitability of these surveillance systems to support predictive analysis for influenza short‐term forecasts at various levels from community to hospitalization and death. An in‐depth review of established frameworks, including those proposed by the Centers for Disease Control and Prevention (CDC), the WHO, and other studies, was conducted to identify structured criteria for assessment [3, 9, 10, 11, 12, 13, 14, 15, 16] (Appendix S2).

Eight key attributes—timeliness, sensitivity, specificity, representativeness, coverage, robustness, completeness, and historical data—were selected from 16 data quality‐related attributes, drawn from a larger pool of 31 attributes (Appendix S2). Attributes were selected based on their alignment with machine learning requirements, their relevance to AI/ML applications, their consistent use in existing surveillance evaluation frameworks, and background knowledge of the expert panel [3, 9, 10, 11, 12, 13, 14, 15, 16]. The expert panel included epidemiologists, public health specialists, virologists, and data scientists and reached consensus through iterative discussions, ensuring balance between AI/ML suitability and epidemiological and public health considerations. This multidisciplinary approach ensured that the attributes were evidence‐based, practical, and relevant to the study's predictive modeling objectives.

Each surveillance system was assigned scores for each attribute based on Phase 1 data and Phase 2 criteria. The scores were weighted using predefined multipliers to reflect the importance of each attribute for training or short‐term forecasting. Weighted scores were averaged to quantify system alignment with predictive model requirements. Two primary metrics were defined to assess system utility:

- **Usefulness for training:** This metric evaluates how well surveillance systems support ML model training by prioritizing the availability of historical data, along with sensitivity, specificity, and completeness. These attributes give models high predictive accuracy generalizable to the wider population under surveillance.
- **Usefulness for short‐term forecasting:** Short‐term forecasting, which spans approximately 1 to 4 weeks ahead, relies on real‐time or near real‐time data and prioritizes timeliness along with robustness, sensitivity, and specificity to provide outputs with high predictive accuracy in real‐time or near real‐time.

Detailed process, definitions, explanations, and the five‐level ranking system for each attribute are provided in Appendix S3.

## 3 Findings

Our research identified 10 surveillance systems that provide data on influenza in New Zealand (Table 1) across four levels (from community to mortality). Strengths and limitations of these systems were assessed across eight critical attributes. They were then evaluated for training and short‐term forecasting applications, respectively (Table 2).

**TABLE 1:** Evaluation of New Zealand national influenza surveillance systems based on key attributes for short‐term forecasting.

Level	Surveillance system	Timeliness	Sensitivity	Specificity	Representativeness	Coverage	Robustness	Completeness and linkability to additional data	Historical data
Community‐based	HealthStat [20, 21, 22, 23, 24, 25, 26]	Moderately high; near real‐time data as monitors people who have presented to their GP with ILI in the past week.	Moderate; does not include virological samples and does not monitor non‐COVID‐19 ILI and COVID‐19 coded consultations.	Moderately low; potential bias due to the voluntary nature of participation and variability in ILI coding by GPs.	Moderate; limited to participating practices and monitors the number of people who consult GPs with an ILI.	Moderate; covering an expanded network of about 400 practices.	Moderate; depending on the consistency of GP reporting.	Moderate; captures data from existing electronic health records from GP.	Moderately high; from 2005 up to now (19 years) history of data collection.
	Healthline [22, 27, 28, 29]	High; real‐time.	Moderately low; depends on self‐reported symptoms and may lack clinical confirmation.	Moderately low; due to a broad definition of ILI, the count of ILI‐related calls may be overestimated. Different ILI definition than the WHO which is used in most influenza surveillance systems globally. Potential bias due to the voluntary nature of participation. Potential biases due to self‐reporting and selective use of the service.	Moderate; although it captures a wide range of individuals with ILI symptoms who may not visit GPs.	Moderately high; wide reach, capturing the number of individuals who may not visit GPs.	Moderate; daily count of all phone calls from individuals with symptoms of any illness triaged for ILI.	Moderately low; depending on the information provided by callers.	Moderately high; from 2000 up to now (24 years) history of data collection.
	FluTracking [5, 30, 31, 32]	Moderately high; near real‐time as weekly data collection allows for timely analysis.	Moderate; can vary in terms of data completeness, accuracy, and representativeness, depending on voluntary participation and the willingness of individuals to report their symptoms.	Moderately low; it is a self‐selected cohort of participants which may cause a form of self‐selection bias. The data may be subject to self‐reporting bias, where participants might underreport or overreport symptoms. Lack of clinical confirmation.	Moderate; includes a diverse group of participants across the country.	Moderately high; covers a wide geographical area with a broad participant base, nationally.	Moderate; relies on continued participant engagement, which can fluctuate.	Moderately low; participation is voluntary, so not all cases are captured. Cannot be easily linked to other datasets via patient identifiers.	Moderate; has been in operation for several years from 2018 up to now (6 years).
	Southern Hemisphere Influenza and Vaccine Effectiveness Research and Surveillance (SHIVERS) [5, 33, 34, 35]	Moderately high; near real‐time data, as virology results take almost a week.	High; robust research methodologies, and laboratory confirmation.	High; provides laboratory‐confirmed cases.	Moderate; findings may not be fully generalizable to the national population.	Moderate; limited to a specific region	Moderately high; the weekly surveys are done during the influenza season.	High; extensive linkages to clinical and lab datasets.	Moderate; longitudinal data from 2018 to 2028 (10 years) is available within the studied population.

	National sentinel general practice‐based Influenza surveillance [5, 22, 23, 24, 25]	Moderately high; near real‐time data, as virology results take almost a week.	Moderate; may overlook mild cases that do not present in general practices, and not consistently capture all ILI cases since 2020.	Moderately high; cases are confirmed by clinical diagnosis, but there is potential for inclusion of non‐influenza respiratory illnesses. Virology testing is employed to confirm the presence of influenza.	Moderate; covers a significant proportion of GPs across the country, but not all regions are equally represented.	Moderate; includes a significant number of patients but misses those who do not visit a GP.	Moderately high; reliable but dependent on GP participation.	Moderately high; can be linked to other datasets via patient identifiers.	High; from 1989 up to now (35 years) history of data collection, though significantly limited since 2020 due to COVID‐19.
Google Flu Trends [36, 37, 38]	High; real‐time estimates of influenza activity based on real‐time search data.	Moderately low; based on search terms, which may not correlate with actual influenza cases.	Low; accuracy can be affected by non‐influenza‐related search behavior. Many search queries could relate to influenza‐like symptoms, not influenza itself.	Moderate; based on search behavior, which may not represent all populations. Less accessible for Low socioeconomic households. Also, people in New Zealand tend to use Healthline before thinking of performing Google searches.	Moderately high; broad coverage due to high internet usage.	Low; the system was eventually discontinued.	Low; cannot be linked to other datasets but could be a supplementary system for traditional surveillance methods.	Low; discontinued (2008–2015 [7 years]) and thus lacks recent data, with limited historical depth.
Hospital‐based	Severe acute respiratory infection (SARI) [5, 22, 24, 25]	Moderately high; near real‐time data, as virology results take almost a week. Also, admissions are accessible daily, but the length of stay, ICU admission and virology results take time to be updated in the dataset.	Moderate; focuses on severe, confirmed respiratory cases. May miss influenza cases with atypical symptoms or misclassify them as other respiratory conditions.	High; data are based on laboratory‐confirmed diagnoses.	Moderate; limited to hospital settings. Hospital‐based data may not capture less severe cases. Also depends on the workload of hospitals, nurses, and laboratories, particularly during peak seasons.	Moderate; limited only at ADHB and CMDHB. Coverage of major hospitals, but not comprehensive. May prioritize other severe respiratory conditions, leading to potential under‐coverage of severe influenza cases.	High; stable, well‐maintained system. Also depends on the workload of hospitals, nurses, and laboratories, particularly during peak seasons.	Moderately high; provides data on influenza‐related complications, virology results for confirmation, and details on current symptoms and chronic medical conditions. Can be linked to other datasets through the NHI. For severe influenza cases, it may shift focus to other respiratory illnesses, which could limit the completeness of influenza‐specific data.	High; longitudinal data are available from Apr 30, 2012, up to now (12 years) within the studied population.
	National minimum data set (NMDS) [5, 22, 25, 39]	Low; not real‐time. Data updates almost monthly may be more delayed for private hospitals.	Moderate; captures a wide range of hospital admissions but may miss severe influenza cases due to less specific targeting of influenza‐related admissions.	Moderately high; data includes detailed diagnoses (ICD codes) and laboratory confirmation of severe influenza cases, although some cases may lack specific coding for influenza, which could reduce accuracy.	High; a wide range of health data from various healthcare settings nationwide offers a comprehensive view of patient care and outcomes.	High; comprehensive and year‐round surveillance of influenza strains, including initial typing and sub‐typing	High; longstanding, stable system.	High; cleaned and high‐quality data are available annually and easily linkable to other health datasets.	High; from 1999 up to now (25 years) history of data collection.
Laboratory‐based	Laboratory‐Based Surveillance (The National Influenza Center at PHF (formerly known as ESR)) [5, 22, 25, 40]	Moderately high; near real‐time data, as virology results take almost a week.	Moderate; detects and characterizes influenza strains with high accuracy. However, clinician‐ordered testing may result in missed true positives. Slightly lower for influenza due to clinician‐ordered testing, which may miss mild cases and reduce capture of the full influenza spectrum.	Moderately high; although specimens are provided from sentinel GP‐based surveillance, it focuses mainly on hospital‐based SARI surveillance potentially biases the data towards more severe cases and underrepresents mild or asymptomatic infections.	Moderately low; based on samples received from healthcare settings, nationally. Mostly covering hospital‐reported severe cases, yet slightly lower for influenza since testing requires clinician order, underrepresenting influenza cases occurring in the community.	Moderate; collected from hospitals and through community‐based laboratories. Coverage for influenza is moderate, as milder cases are managed outside hospitals leading to gaps in community‐based influenza coverage.	High; well‐established and nationally recognized lab.	Moderately high; detailed and accurate pathogen data can be linked with hospital and GP datasets.	High; from 1990 until now (34 years) history of data collection.
Mortality	Mortality Datasets from the Ministry of Health [5, 22, 41]	Not real‐time; data are delayed due to the death registration and certification processes.	Moderately low; captures all registered deaths. But moderately low sensitivity for influenza since death records often prioritize other underlying conditions over influenza‐specific causes.	High; causes of death are determined through medical certification, using ICD‐coded data.	Moderate; nationwide, covering all deaths. As it may focus on severe cases, potentially underrepresenting influenza‐related deaths occurring in the broader community.	Moderate; comprehensive dataset covering national mortality data. It may miss nonhospitalized or mild cases resulting in death outside of hospital settings.	High; a longstanding system with High‐quality, verified data suitable for trend analysis.	High; high‐quality data including demographics and ICD‐coded data, linkable with other health datasets using NHI.	High; from 1988 up to now (36 years) history of data collection.

**TABLE 2:** Comprehensive evaluation of the usefulness of New Zealand national influenza surveillance systems for training and short‐term forecasting.

Attributes	Multiplier for training^a^, ^b^	Multiplier for short‐term forecasting^c^, ^d^	Community						Laboratory^e^	Hospital		Mortality
			HealthStat	Healthline	FluTracking	SHIVERS	National sentinel GP‐based influenza surveillance	Google Flu	Laboratory‐based surveillance NIC	SARI	NMDS	Mortality
Timeliness	0.05	0.25	4	5	4	4	4	5	4	4	1	1
Robustness	0.10	0.15	3	3	3	4	4	1	5	5	5	5
Sensitivity	0.15	0.15	3	2	3	5	3	2	3	3	3	2
Specificity	0.17	0.15	2	2	2	5	4	1	4	5	4	5
Historical data	0.18	0.05	4	4	3	3	5	1	5	5	5	5
Representativeness	0.10	0.10	3	3	3	3	3	3	2	3	5	3
Coverage	0.10	0.05	3	4	4	3	3	4	3	3	5	3
Completeness	0.15	0.10	3	2	2	5	4	1	4	4	5	5
Usefulness for training^a^	The summation of multiplier for training equals 1	Not applicable	3.06	2.91	2.83	4.09	3.83	1.85	3.83	4.10	4.33	3.95
Usefulness for short‐term forecasting^c^	Not applicable	The summation of multiplier for short‐term forecasting equals 1	3.15	3.20	3.05	4.20	3.75	2.50	3.80	4.05	3.55	3.25

*Note:* Green: Above the moderate (≥ 3) for intermediate and critical attribute, plus high scores overalls, indicating strong suitability for training and/or short‐term forecasting. Priority bands for the attributes for that specific application; low priority (< 0.125), intermediate priority (≥ 0.125 and < 2.5), and critical priority (≥ 2.5).

^a^ Training is defined as the process where an algorithm learns to map input data to desired outcomes by optimizing itself using provided examples, enabling it to generalize to unseen data.

^b^ Weight assigned to each attribute for evaluating the system's suitability for training ML models.

^c^ Short‐term forecasting spans over days or weeks (1 to 4 weeks ahead), based on real‐time or near real‐time data.

^d^ Weight assigned to each attribute for evaluating the system's utility for short‐term forecasting.

^e^ Laboratory‐based surveillance is defined as a separate category. While it primarily captures severe hospital cases, it also includes data from community sources, such as SHIVERS and general practice sentinel surveillance.

Community‐based systems, such as HealthStat and FluTracking, demonstrated strong timeliness, necessary for short‐term forecasting (scores: 3.15 and 3.05). However, their reliance on voluntary self‐reporting of symptoms results in lower specificity that limits their effectiveness in training ML models. Healthline, though real‐time, faced similar constraints because it relies on self‐reported symptoms and uses a broader ILI definition than the WHO standard (see Appendix S1). This broader definition does not require fever and may capture many non‐influenza illnesses, which reduces its influenza virus‐specific accuracy. Among community systems, Southern Hemisphere Influenza and Vaccine Effectiveness Research and Surveillance (SHIVERS) stood out with the highest training (4.09) and short‐term forecasting (4.20) scores. Its influenza virus‐specific laboratory‐confirmed data provide sensitivity, specificity, and historical depth, making it highly effective for both applications.

Hospital‐based systems scored highly in both categories. SARI achieved the highest short‐term forecasting score (4.05) and strong training performance (4.10), due to its available longitudinal data, sensitivity, and specificity. The National Minimum Data Set (NMDS), although useful for training (4.33) due to its extensive historical data, was less suitable for short‐term forecasting because of its limited timeliness. Laboratory‐based surveillance, represented by the National Influenza Center, bridges community and hospital data, offering moderate utility for both training and short‐term forecasting. Although not the highest performer, its role in integrating data from various sources improves its utility.

The mortality dataset has historical depth but is constrained by poor timeliness, making it unsuitable for short‐term forecasting. Nonetheless, it remained valuable for long‐term trend analysis.

SHIVERS and SARI emerged as the most useful systems for community and hospital settings, respectively. These results highlight the complementary roles of different systems in supporting AI/ML applications.

## 4 Discussion

This study evaluates the suitability of New Zealand's influenza surveillance systems for predictive models, focusing on training and short‐term forecasting capabilities. While systematic reviews have looked at general data quality, to our knowledge, this is the first study looking at which surveillance system attributes matter for AI/ML applications, bringing together both public health and data science perspectives [42]. Although we acknowledge that each surveillance system is designed to meet its own objectives, the growth of AI/ML in health decision making highlights the need to consider how these systems might also support predictive analytics and early‐warning applications.

For predictive models, the quality of input data is as important as the model design [18, 43]. To ensure outputs achieve optimal predictive accuracy, models must be trained with sufficiently well‐characterized data that meet specific criteria of usefulness for training [18, 43]. Selecting an optimal combination of data sources therefore contributes to effectively supporting influenza forecasting. To the best of our knowledge, previous influenza forecasting studies have used single data sources, occasionally supplemented by laboratory data, and typically integrating search engines or social media data [44, 45, 46]. In New Zealand, the SARI dataset has already been integrated for ML‐based short‐term forecasting, and the impact of integrating laboratory data was investigated [47]. While individual systems exhibit distinct strengths, leveraging a combination of community, laboratory, and hospital‐based surveillance systems could optimize forecasting accuracy [19, 44, 48]. Evidence from prior studies shows integrating syndromic and laboratory‐confirmed data enhances model accuracy, supporting a multisystem approach [44].

It is possible that the predictive value could be improved by using multiple surveillance systems instead of one. In New Zealand, influenza surveillance covers different levels of severity. Using SHIVERS for community‐level data and SARI for hospital‐level data could enhance the predictive value of models for forecasting influenza burden and support effective resource allocation. Also, combining syndromic and etiological surveillance systems at different stages—using the high accuracy of etiological data to train machine learning models and the real‐time capabilities of syndromic data for short‐term forecasting—may enhance predictive value.

This study also suggests reviewing systems with lower scores to determine whether they need to be improved, integrated into other data sources, or discontinued based on their respective objectives and cost‐effectiveness. Such a review could increase their value for training and short‐term forecasting, improve resource use, and help preserve valuable data sources.

Additionally, the NMDS dataset showed strong training potential due to its historical depth. Future research could explore its forecasting capability, and if predictions were accurate, improving its timeliness to make it a near real‐time system could be justified.

Another key implication of this study is the need to address the underrepresentation of certain populations in existing surveillance systems. This gap may limit the accuracy and generalizability of predictive models and subsequent public health decisions. Integrating lower‐cost syndromic surveillance systems like FluTracking with other data sources, particularly systems providing etiological laboratory data, could strengthen forecasting capabilities. Such enhancements would support more comprehensive early detection and prediction of surges in influenza infection at both community and hospital levels.

The evaluation framework developed in this study allows comparisons between real‐world surveillance systems, defining a hypothetical optimal data source for predictive model training and short‐term forecasting. By identifying characteristics key to predictive models, the framework could inform strategic data collection efforts and surveillance investments, enhancing the usefulness of forecasting with better data quality.

In future research, we aim to develop a training dataset aligned with different levels of influenza severity, incorporating well‐suited data sources to enhance ML‐based forecasting models. A promising approach is the application of transfer learning within a multivariate framework to integrate time series data across severity levels. This approach could make models leverage cross‐learning dependencies, improving forecasts in multivariate‐to‐multivariate models (e.g., DeepAR) or multivariate‐to‐univariate models (e.g., temporal fusion transformer). Additionally, instead of traditional ensemble methods, forecasting could be strengthened by training specific models on diverse datasets, capturing distinct features of influenza activity to improve predictive accuracy.

This study has limitations. Although the attribute rankings of surveillance systems in this study were validated by co‐authors with expertise in epidemiology, virology, public health, data science, and machine learning, the subjective nature of these evaluations may limit the validity and generalizability of the conclusions. We have provided transparent reasoning and justifications to allow critical review and further refinement of the evaluation framework. Also, sensitivity and specificity were not measured directly but were instead inferred from proxy indicators like laboratory confirmation and the use of case definitions. Moreover, the findings of this study raise several hypotheses, particularly regarding multisource datasets integrating syndromic and etiological data from different levels of severity, warranting further practical investigations to assess their feasibility and explore their implications for resource planning and decision making.

## 5 Conclusions

This study proposes an evaluation framework for assessing the suitability of surveillance systems for modeling future short‐term disease scenarios for influenza. It emphasizes their alignment with predictive model requirements, notably for training and short‐term forecasting. Key findings demonstrate the strengths of specific systems, which in New Zealand include SHIVERS and SARI, which perform well in community and hospital settings, respectively. The framework lays the groundwork for more accurate and timely forecasting by providing a foundation for creating datasets aligned with AI/ML techniques, integrating multisource datasets, and addressing gaps in representativeness. This multidisciplinary study not only advances academic understanding but also ensures that public health surveillance systems can be improved to better support measures to reduce the burden of influenza.

## Author Contributions

**Negin Maroufi:** conceptualization, writing – review and editing, methodology, writing – original draft, formal analysis. **Michael G. Baker:** supervision, methodology, validation, review and editing. **Lucy Telfar Barnard:** supervision, validation, methodology, review and editing. **Qiu Sue Huang:** supervision, methodology, validation, review and editing. **Gillian Dobbie:** supervision, methodology, validation, review and editing. **Nayyereh Aminisani:** methodology, visualization, review and editing. **Steffen Albrecht:** methodology, validation, review and editing. **Nhung Nghiem:** methodology, visualization, review and editing.

## Ethics Statement

This study did not require ethics, as it did not involve human participants or the use of personal data. The analysis was based on data from existing surveillance systems and publicly available information, ensuring compliance with ethical guidelines.

## Conflicts of Interest

The authors declare no conflicts of interest.

## Supporting information


**Appendix S1:** Influenza Surveillance Systems in New Zealand.

**Appendix S2.** Summary of Surveillance System Features, Their Definitions, and Supporting References.

**Appendix S3.** Attribute Definitions and Ranking Criteria for Evaluating Surveillance System Suitability for Training and Short‐term Forecasting.

## Data Availability

The data that supports the findings of this study are available in the supplementary material of this article.
