# Supplementary material for: A Framework for Evaluating the Use of Surveillance Systems for Short‐Term Influenza Forecasting
Source: Influenza Other Respir Viruses. 2025 Jul 29;19(8):e70144. doi: 10.1111/irv.70144 (PMC12307093; doi:10.1111/irv.70144)
Supplement: Supplementary file 1 — Appendix S1: Influenza Surveillance Systems in New Zealand. [file IRV-19-e70144-s003.docx]

# Appendix 1: Influenza Surveillance Systems in New Zealand

## Community-based surveillance

### HealthStat

In late 2005, HealthStat was launched and has since been utilized for national public health surveillance in New Zealand, particularly for monitoring influenza-like illness (ILI) and gastroenteritis in individuals attending general practices ^1-3^. Covering approximately 1.7 million patients weekly, HealthStat provides district health boards (DHBs) with valuable insights into public health trends at the DHB level, enabling near real-time assessments of population health needs ^1^. ILI-related data is collected nationally from around 400 participating general practices on a weekly basis, with an option for more frequent, daily monitoring if necessary ^1,4^. The case definition for ILI follows the criteria of an acute upper respiratory tract infection, characterized by the sudden onset of two or more symptoms, including chills, fever, headache, and muscle aches (myalgia) ^1,5,6^. To identify ILI-related consultations, including both COVID-19 and non-COVID-19 cases, SNOMED and Read codes are used ^1^. SNOMED is an internationally recognized clinical terminology system used to standardize health information across different systems, while Read codes are a coding system historically for classifying clinical terms in patient records ^7^. However, HealthStat does not include virological surveillance, meaning that patients presenting with ILI are not routinely tested with swabs to confirm the presence of influenza or other respiratory viruses ^1,6^. The data is only shared with DHBs with the consent of the participating practices and is also provided to the New Zealand Institute for Public Health and Forensic Science (formerly the Institute of Environmental Science and Research (ESR)) on a weekly basis ^1^.

### Healthline

Healthline provides round-the-clock access to free health advice, information, and treatment from healthcare professionals ^3,8-10^. Funded by Health New Zealand in collaboration with The Māori Health Authority and operated by New Zealand Telehealth Services, Healthline monitors and triages calls using electronic clinical decision support software ^3,8^. Symptomatic calls related to acute respiratory infections (ARI) such as colds, flu, coughs, and sore throats are categorized as influenza-like illness (ILI), while non-symptomatic inquiries are excluded ^8,9^. This contributes valuable data for public health surveillance; however, Healthline operates as a syndromic surveillance system, focusing on the monitoring of symptoms rather than virological testing. Unlike the WHO definition of ILI, which requires the presence of fever, Healthline uses a broader definition that does not require fever for categorization ^8,9^. This broader definition captures less severe symptoms, resulting in a large volume of ILI-related calls each year ^8^. Trends observed in Healthline data may also be influenced by increased public awareness of specific illnesses, including those highlighted by media reporting ^3,8,10^.

### FluTracking

Unlike traditional sentinel systems, FluTracking is an online health participatory surveillance system that monitors influenza-like illness (ILI) symptoms in the communities of Australia and Aotearoa New Zealand ^11-14^. Participants, including members of the public, voluntarily report their symptoms weekly ^12,14^. Developed by Hunter Population Health in collaboration with The University of Newcastle, FluTracking has been operational in New Zealand since its launch in 2018 ^14^. Over 124,000 participants have registered with FluTracking, covering more than 1,000 postcodes in New Zealand ^14^. It provides near real-time data, as participants submit weekly reports for themselves or on behalf of their family members, allowing for timely monitoring of ILI trends ^12,14^.

At initial enrolment, demographic information such as month and year of birth, sex, ethnicity, location (postcode of residence), the highest level of education (for participants aged 15 years and older), and details about COVID-19 vaccination (number of doses received, and the month and year of the latest dose) are collected ^12,14^. Registered participants receive a weekly email asking them to report any cold, flu, or COVID-like symptoms experienced in the previous week ^12,14^. Symptoms tracked include the presence or absence of cough, fever, sore throat, shortness of breath, runny nose, changes in sense of taste or smell, and headache ^12,14^. If individuals experience symptoms, they are asked additional questions about whether they sought healthcare, missed work or normal duties due to the illness, or were tested for COVID-19 or influenza elsewhere ^12-14^. Information on vaccination status for COVID-19 and the annual influenza vaccine is also collected, along with testing for COVID-19 in the previous week^12-14^.

Although participatory health surveillance systems like FluTracking provide valuable community-level data for tracking outbreaks of infectious diseases, this system does not include systematic testing to confirm whether the illness is attributable to influenza infection, which limits the ability to estimate the positive predictive value ^13,14^. Additionally, due to the voluntary nature of this system, completeness may vary, leading to reporting bias ^12-14^. The system may be biased in terms of survey participants and response rates over time and may not equally represent all population segments, such as individuals with lower socio-economic status or minority ethnic groups (self-selection bias) ^12-14^. Raw data from FluTracking New Zealand can be obtained through the New Zealand Ministry of Health ^12-14^.

### Sentinel General Practice-Based Influenza Surveillance

This surveillance system collects data on respiratory viruses circulating in the community and has contributed to the WHO Global Influenza Programme since 1989 ^6,13^. It is coordinated nationally by the New Zealand Institute for Public Health and Forensic Science (formerly the Institute of Environmental Science and Research (ESR)) on behalf of the Ministry of Health and supported locally by public health service surveillance coordinators ^6,13^. These coordinators recruit general practices across regions on a voluntary basis, aiming for coverage proportional to the district health board population (approximately one practice per 50,000 residents) ^3,6,13^. The system typically operates from May to September and defines influenza-like illness (ILI) as a sudden onset of upper respiratory symptoms accompanied by at least two of the following: fever, chills, headache, or muscle aches ^6^. Participating GPs report weekly ILI consultations by age group using a standardized form ^6^. Additionally, they collect respiratory samples—usually nasopharyngeal or throat swabs—from the first ILI patient seen each Monday to Wednesday, with larger practices submitting up to six samples per week ^6^. These are sent to PHF’s (formerly known as ESR) National Influenza Center or regional hospital laboratories in Auckland, Waikato or Christchurch for testing for influenza, SARS-CoV-2, RSV, and other respiratory viruses ^3,4,6,13^. While laboratory testing has continued, regular ILI reporting by GPs has declined since the COVID-19 pandemic due to increased pressure on primary care, and participation levels have remained lower in the years following ^5,6,13^.

### Southern hemisphere influenza and vaccine effectiveness research and surveillance (SHIVERS)

A multi-agency and multi-disciplinary project led by PHF (formerly known as ESR) aims to assess the disease burden, epidemiology, causes risk factors, immune response, and effectiveness of vaccination and prevention strategies for influenza and other respiratory diseases ^13,15^. From 2012 to 2017, SHIVERS conducted community-based sentinel general practice surveillance and one-year-only community cohort ^13,16^. Since 2018, this initiative has expanded into the WellKiwis study, a longitudinal cohort study that will run until 2028 ^13,16^. The project involves pre- and post-season blood collection and the completion of weekly online surveys during the flu season. These surveys capture influenza-like illness (ILI) or acute respiratory illness (ARI) symptoms, and swabs are collected from individuals who meet the criteria ^16,17^.

This community-based research, focused in Wellington, offers robust surveillance outputs ^15,16^. Due to the weekly collection of data and the time required for testing specimens, it can be considered a near real-time system ^15,16^. While it provides high-quality data from a well-defined cohort, its representativeness may be limited ^13,15,16^. Additionally, maintaining this cohort is resource-intensive and requires significant funding and coordination ^13,15,16^. Despite these challenges, the project's comprehensive approach and detailed data collection contribute valuable insights into the epidemiology and prevention of respiratory infectious diseases ^13,15,16^.

### Google Flu Trends (GFT)

Google Flu Trends (GFT) was an innovative influenza surveillance system that utilized search query data to estimate influenza activity in real-time, conducted globally including New Zealand from 2008 to 2015 ^18,19^. By analyzing the frequency of specific flu-related search terms entered by users in New Zealand, GFT aimed to provide early detection of influenza outbreaks and track the spread of the virus ^18-20^. This approach offered a complementary perspective to traditional surveillance methods, leveraging the vast amount of data generated by internet users ^18,19^. However, while GFT demonstrated potential for rapid, real-time surveillance, its accuracy and reliability were sometimes questioned due to fluctuations in search behavior unrelated to actual influenza activity ^19^. Despite these limitations, GFT highlighted the potential for digital epidemiology to enhance public health surveillance systems ^18,19^.

## Hospital-based and Mortality Surveillance

### Severe Acute Respiratory Infection (SARI) Surveillance

The hospital-based surveillance of Severe Acute Respiratory Infection (SARI) serves as a national routine dataset for monitoring trends in the epidemiology and outcomes of respiratory ailments among hospitalized patients in New Zealand ^3,5,13^. Initially funded by the US Centers for Disease Control and Prevention (CDC) under SHIVERS project until 2016, the New Zealand Ministry of Health has sustained funding for hospital-based SARI surveillance since 2017 ^3,5^.

This surveillance initiative encompasses patients admitted overnight with Acute Respiratory Illness (ARI) across four hospitals: Auckland City, Starship, Middlemore, and KidzFirst, which cater to central, eastern, and southern Auckland, specifically the Auckland District Health Board (ADHB) and Counties Manukau District Health Board (CMDHB) ^3,5,6,13^. If a patient meets the Severe Acute Respiratory Illness (SARI) case definition—an acute respiratory illness with a history of fever or measured fever of ≥38˚C, cough, onset within the past 10 days, and requiring inpatient hospitalization—a case-report form is completed ^5,6,13^. This form includes the patient's demographic information, presenting symptoms and illness, pre-hospital healthcare, medication usage, influenza vaccination history, co-morbidities, disease course and outcomes (including major treatments, ICU admission, and mortality), epidemiologic risk factors, and laboratory results. Additionally, a specimen is collected to test for influenza and other respiratory pathogens (including rhinovirus, enterovirus, adenovirus, human metapneumovirus, parainfluenza virus (types 1-3), RSV, and SARS-CoV-2). Specimens are also collected for some non-SARI cases (i.e. acute respiratory illness patients not meeting SARI case definition) ^5,6,13^. While the testing panel for SARI cases has remained consistent since 2012, the selection of viruses tested for non-SARI cases varies and is influenced by several factors, including clinical decision-making, laboratory capacity, and patient characteristics ^5,6,13^.

### National Minimum Data Set (NMDS)

The National Minimum Data Set (NMDS) is a hospital-based surveillance system in New Zealand that contains a comprehensive collection of hospital discharge information from both public and private hospitals nationwide ^3,21^. It is funded and managed by Health New Zealand and serves a variety of stakeholders, including Health Districts, Primary Health Organizations, clinicians, researchers, and the general public ^3,21^. Established in 1993, the current version of the NMDS was introduced in 1999. The dataset includes detailed ICD-coded clinical data from 1988 to the present ^3,6,13,21^. Data from public hospitals are loaded within 21 days after discharge, and an annual report is accessible through an online web tool ^3,21^.

### Mortality Dataset

This dataset provides information on demographics and the underlying causes of all deaths based on registered deaths in New Zealand ^3,22^. It is sourced from the mortality collection and publications in the Ministry of Health Mortality and Demographic Data series and is managed by Health New Zealand ^3,22^. The number of deaths and trends are available from 1948, with detailed data on causes of death available from 1988 onwards ^22^. Data for 2020 is provisional and for 2021 is preliminary, due to pandemic-related delays in the coronial confirmation of the cause of death ^22^. Although the data is not available in real-time due to the time required for verification and processing, it is of high quality ^13,22^. The primary limitation is the lag between the occurrence of deaths and the availability of data for analysis ^22^.

## Laboratory-Based Surveillance

A critical component of the public health infrastructure in New Zealand, aimed at monitoring and identifying pathogens responsible for various respiratory infectious diseases, including influenza, is the laboratory-based surveillance system ^3,6,13,23^. Managed by the New Zealand Institute for Public Health and Forensic Science (formerly the Institute of Environmental Science and Research (ESR)), this system relies on contributions from several regional virus diagnostic laboratories located in hospitals across Auckland, Middlemore, Waikato, Wellington, Christchurch, and Dunedin ^13,23^. These laboratories report viral identifications, including influenza, largely from outpatient clinics and inpatient hospitals during routine laboratory diagnostic investigation, to the National Influenza Center on a weekly basis. The Center then compiles and disseminates virology surveillance data nationwide ^3,13^. In addition, this laboratory network conducts testing for public health surveillance including hospital-based SARI and sentinel GP-based surveillance and SHIVERS community cohort study. While the system provides valuable data year-round and has a longitudinal history, it has limitations; specifically, it only includes positive virus reports, making it challenging to derive trends in terms of the proportion of positives. Additionally, most testing is initiated and based on clinicians' judgment rather than a systematic public health surveillance approach. Despite these limitations, the data remains crucial for detecting outbreaks, guiding treatment protocols, and assessing the effectiveness of public health interventions ^3,13,23^.

**Table 1. An Overview of National Influenza Surveillance Systems in New Zealand**

| Surveillance System | Description | Available Years of Data | Community or hospital-based | Real-time Capabilities | Data Quality | Data Collection | Strengths | Limitations |
| --- | --- | --- | --- | --- | --- | --- | --- | --- |
| HealthStat | Computer-based ILI surveillance based on a national collection of data from general practices using electronic health records. | 2005 – up to now | Community-based surveillance, through GPs. | Near real-time data as monitors people who have presented to their GP with ILI in the past week. | Can vary depending on the consistency of GP reporting. | Utilizes existing electronic health records from GP and is only shared with DHBs with the explicit consent of the participating practices. | Expanded network of about 400 practices.  Access to near real-time primary care data analysis.  Monitors the number of people who consult GPs with an ILI.  Can provide detailed clinical data. | Does not include virological samples.  Does not monitor non-COVID-19 ILI and COVID-19 coded consultations.  ILI consultation based on coding can produce variation by GPs because of various interpretations. |
| Healthline | Free national 24/7 telephone-based health advice service that collects data on ILI from callers. | 2000 – up to now | Community-based surveillance | Real-time | Can vary in terms of data completeness, accuracy, and representativeness, depending on the information provided by callers. | Daily count of all phone calls from individuals with symptoms of any illness triaged for ILI. | Wide reach, capturing a number of individuals who may not visit GPs. | Data quality can vary based on caller self-reporting.  Due to a broad definition of ILI, the count of ILI-related calls may be overestimated.  Different ILI definition than the WHO which is used in most influenza surveillance systems globally.  Does not include details of the individuals and specific symptoms  Does not include virological samples. |
| FluTracking | Online self-reporting respiratory illness surveillance system. | 2018 – up to now | Community-based surveillance | near real-time data, as participants submit their reports weekly. | Can vary in terms of data completeness, accuracy, and representativeness, depending on voluntary participation and the willingness of individuals to report their symptoms. | *Initial registration:*   - Month and year of birth - Sex - Receipt of influenza vaccine in the preceding year - Number of COVID-19 vaccination doses received, and the month and year in which the latest dose was received. - Postcode of residence - First Nations status (AU) or ethnicity (NZ) - Face-to-face contact with patients (for participants 15 years or older) - Highest level of education (for participants 15 years or older)   *Weekly surveys:*   - Fever - Cough - Sore throat - Runny nose - Shortness of breath - Any change in sense of taste or smell - Headache   If the answer to a symptom is ‘yes’, follow-up questions will be:   - Number of days absent from work or normal duties. - Visits to healthcare providers - Results of test for Influenza or COVID-19 - Any subsequent COVID-19 vaccines - Receipt of influenza vaccine in the current year | Not a traditional sentinel system (using electronic systems).  Providing valuable community-level data on influenza and COVID-like illness.  Real-time syndromic surveillance data on ILI symptoms, allowing for timely monitoring of ILI trends. | The self-selected cohort of participants may cause a form of self-selection bias.  The data may be subject to self-reporting bias, where participants might underreport or overreport symptoms.  No virology tests are available to confirm. |
| Southern Hemisphere Influenza and Vaccine Effectiveness Research and Surveillance" (SHIVERS) | SHIVERS is a research program collecting detailed data on influenza, including virus characteristics and vaccine effectiveness. | 2018 – up to now (ends in 2028) | Community-based surveillance | ARI/ILI syndromic surveillance is real-time, while laboratory confirmation for influenza and COVID-19 is near real-time, with virology results typically available within 24 to 48 hours. | High, due to the research methodologies, and laboratory confirmation. | *Demographics:*   - NHI - Full Name - Enrolment date - Household type - Date of birth - Sex - Ethnicity - Education - Employment status - NZDep   *Housing and other risk factors:*   - Number of individuals in the household - Number of bedrooms - Home status in winter - Houses have mold or damp or smell musty - Living with smoker - Smoking status - Health status - BMI - Underlying conditions   *Vaccination:*   - Flu vaccination (including history, date) - Post-vaccination blood (date) - History of COVID-19 Vaccination (dates)   *Weekly surveys:*   - Fever - Cough - Sore throat - Runny nose - Wheezing - Headache - Muscle ache - Congestion - Shortness of breath - Sputum/Phlegm - Loss of taste or smell - Diarrhea - Vomiting - Unexplained changes in sleep - Unexplained changes to feeding - Irritability - Rash - Any other symptoms and severity - Nurse review | The largest and most comprehensive influenza research initiative in the southern hemisphere.  Laboratory confirmation of influenza cases. | Findings may not be fully generalizable to populations.  The weekly surveys are done during the flu season.  May have reported delays for non-influenza respiratory viruses such as respiratory syncytial virus (RSV), rhinovirus, human metapneumovirus (HMPV), parainfluenza virus types 1-3, adenovirus, and enterovirus. |
| National sentinel general practice virological surveillance | A syndromic surveillance system involves selected GPs collecting and reporting data on ILI and sending samples for laboratory testing. | 1989 - up to now | Community-based surveillance | near real-time data, as virology results takes almost a week. | Due to the COVID-19 Pandemic, no cases of ILI symptoms were reported during 2020-21.  Currently, only the virological sampling component of this system is operating.  Can vary depending on the consistency and voluntary of GPs reporting. | *HealthLink e-ILI form:*   - Cough - Measured fever ≥ 38˚C or history of fever. - Days since the start of the symptoms - If patients have had antiviral medication prescribed, and list of medications. - Influenza vaccination status for the current year   If a specimen is collected; the date and type of the specimen | Allows for timely monitoring of influenza trends.  New Zealand has one of the world’s highest rates of the use of Practice Management Systems.  Provides virological data on circulating respiratory viruses. | The data collection for numerators and denominators is manual-based.  Limited coverage may not represent the entire population.  Relies on the voluntary participation of GPs.  Lower levels of participation during the COVID-19 pandemic due to the pandemic’s heavy impact on frontline health workers led this system to lose the measurement for the ILI syndromic disease component. |
| Google Flu Trends | Influenza activity was estimated by analyzing the frequency of flu-related search terms entered by users globally in 25 countries including New Zealand | 2008 - 2015 | Community-based, utilizing data from internet users | Was providing real-time estimates of influenza activity | Low; depends on search behavior, which could be influenced by factors unrelated to actual flu prevalence | Analysis of Google search query data related to influenza symptoms and activity | Providing immediate daily and weekly total data graphs.  Free.  Broad coverage due to high internet usage.  Potential for early detection of outbreaks. | Accuracy can be affected by non-flu-related search behavior.  Not a replacement, but a supplementary system for traditional surveillance methods.  Dependent on the availability of search data.  People in New Zealand tend to use Healthline before thinking of performing Google searches.  Less accessible for Low socioeconomic households. |
| Severe Acute Respiratory Infection (SARI) | Patients with acute respiratory illnesses admitted overnight to any of the four hospitals: Auckland City Hospital and the associated Starship Children’s Hospital, Middlemore Hospital, and the associated KidzFirst Children’s Hospital) are screened by research nurses daily. | Apr 30, 2012 - up to now | Hospital-based surveillance | SARI syndromic surveillance is real-time, while laboratory confirmation for influenza and COVID-19 is near real-time data, as virology results take almost a week. | High-quality data; however data completeness, and representativeness, depend on the workload of hospitals, nurses, and laboratories, particularly during peak seasons.  Data quality is being done under a short questionnaire for each person including NHI check, details on Ethnicity review, and if clinical sample data has been received and its details. | *Essential Data:*   - NHI - Date of birth - Sex - Ethnicity - Auckland residence or not - Center - Admission date - Discharge date - Died while in hospital / Death date - Respiratory admission diagnosis (including history of cough and fever) - Specimen (if it has been taken and the date of it) - Symptoms (including runny nose, sneeze, sore throat, wheeze, short breath, chills, headache, aching muscle, abdominal pain, nausea, vomiting, diarrhea, and any additional signs) - Flu vaccination history - Working in a healthcare setting - Obesity   *Medical Record:*   - Chronic medical conditions - If female, information on pregnancy - ICU admission and discharge date - Therapies | Tracks severe influenza cases requiring hospitalization.  Provides data on influenza-related complications.  Supports pandemic influenza severity assessment.  Provides virology results for confirmation.  Provides details on current symptoms and chronic medical conditions. | Limited to hospital settings.  Limited only at ADHB and CMDHB, therefore is not scalable across New Zealand.  Admissions are accessible daily, but the length of stay, ICU admission and virology results take time to be updated in the dataset.  Requires research nurses to collect data which is resource-intensive and costly. |
| National Minimum Data Set (NMDS) | Present aggregated data from both public and private hospitals, comprising clinical details for admitted patients and those treated daily at the national level. | 1999 – up to now | Hospital-based surveillance | Not real-time. Data updates almost monthly may be more delayed for private hospitals | High | - NHI - Event type code - Event start and end date - Domicile code - Sex - Date of birth - Ethnicity - NZ resident status - Country of birth - Occupation - Detail data on birth such as weight, age of mother, etc. - Detail data on diagnosis such as ICD-code, clinical code, procedures, etc. | Captures a wide range of health data from various healthcare settings, offering a comprehensive view of patient care and outcomes.  It uses the ICD-coded data. | Potential lag in data availability; the cleaned and high-quality data is available annually. |
| Laboratory-Based Surveillance (The National Influenza Center at PHF (formerly known as (ESR)) | Hospital laboratories serve almost 70% of the NZ population. | 1990 – up to now | Both community and hospital-based | near real-time data, as virology results take almost a week. | High; due to being all year-round longitudinal up to now and laboratory confirmation of influenza cases. | Specimens are collected from hospitals and through community-based laboratories. | Detailed and accurate pathogen data.  Essential for outbreak detection.  Year-round surveillance of influenza strains, including initial typing and sub-typing | Specimens from sentinel GP-based surveillance and mainly hospital-based SARI surveillance potentially bias the data towards more severe cases and underrepresent mild or asymptomatic infections.  Clinician-ordered testing is based on clinical judgment, rather than a systematic public health surveillance approach.  Lack of data on negative test results. |
| Mortality Datasets from the Ministry of Health | Present and assess data regarding the underlying causes of all recorded deaths in New Zealand annually. | 1988 – up to now | Community-based; encompassing deaths from all settings. | Not real-time. Data is delayed due to the death registration and certification processes. | High; verified based on death certification and registration processes | *Demographics:*   - Year - Common cause of death - Type - Sex - Ethnicity - Age group - DHB | It uses the ICD-coded data.  Comprehensive coverage of mortality data  High-quality, verified data.  Longitudinal data availability for trend analysis | Potential lag in data availability. |

**References:**

1. HealthStat. HealthStat. <https://www.healthstat.co.nz/>

2. 11. Inﬂuenza. Health New Zealand. <https://www.tewhatuora.govt.nz/for-health-professionals/clinical-guidance/immunisation-handbook/11-influenza/>

3. Immunisation Handbook 2020. New Zealand Ministry of Health - Manatu Hauora. <https://www.health.govt.nz/our-work/immunisation-handbook-2020/11-influenza>

4. Adnan M, Peterkin D, Lopez L, Mackereth G. Electronic Sentinel Surveillance of Influenza-like Illness. *Experience from a pilot study in New Zealand*. 2017/12/20 2017;26(01):97-107. doi:10.4338/ACI-2016-06-RA-0103

5. Huang QS, Baker M, McArthur C, et al. Implementing hospital-based surveillance for severe acute respiratory infections caused by influenza and other respiratory pathogens in New Zealand. *Western Pac Surveill Response J*. Apr-Jun 2014;5(2):23-30. doi:10.5365/wpsar.2014.5.1.004

6. Lopez L, Wood T, Prasad N, Huang S. Influenza Surveillance in New Zealand 2016. 2018;

7. Zealand HN. SNOMED CT. Updated 15 July 2024. <https://www.tewhatuora.govt.nz/health-services-and-programmes/digital-health/snomed-ct-national-release-centre/snomed-ct>

8. Healthline. Health New Zealand. <https://info.health.nz/services-support/health-and-disability-providers/healthline/>

9. Wilson MK, Pienaar F, Large R, Wright M, Todd VF. Enhancing Aotearoa, New Zealand's Free Healthline Service through Image Upload Technology. *Int J Telemed Appl*. 2024;2024:6644580. doi:10.1155/2024/6644580

10. Sharing Excellence in Health and Disability Information Management. Wellington: Ministry of Health. <https://www.health.govt.nz/publication/sharing-excellence-health-and-disability-information-management-2006>

11. Dalton CB, Carlson SJ, Butler MT, Elvidge E, Durrheim DN. Building Influenza Surveillance Pyramids in Near Real Time, Australia. Article. *Emerging Infectious Diseases*. 11// 2013;19(11):1863-1865. doi:10.3201/eid1911.121878

12. Harvey EP, Trent JA, Mackenzie F, Turnbull SM, O’Neale DRJ. Calculating incidence of Influenza-like and COVID-like symptoms from Flutracking participatory survey data. *MethodsX*. 2022/01/01/ 2022;9:101820. doi:<https://doi.org/10.1016/j.mex.2022.101820>

13. Respiratory Surveillance Sentinel Practice Information. Institute of Environmental Science and Research Limited. <https://www.esr.cri.nz/expertise/public-health/infectious-disease-intelligence-surveillance/respiratory-surveillance-sentinel-practice-information/>

14. About FluTracking. Public Health Group from the Ministry of Health of New Zealand. <https://info.flutracking.net/about/>

15. Huang QS, Turner N, Baker MG, et al. Southern hemisphere influenza and vaccine effectiveness research and surveillance. *Influenza Other Respir Viruses*. 2015;9(4):179-190.

16. WellKiwis Influenza Study. Environmental Science and Research (ESR). <https://www.wellkiwis.co.nz/>

17. Huang QS, Bandaranayake D, Wood T, et al. Risk factors and attack rates of seasonal influenza infection: results of the Southern Hemisphere Influenza and Vaccine Effectiveness Research and Surveillance (SHIVERS) seroepidemiologic cohort study. *The Journal of infectious diseases*. 2019;219(3):347-357.

18. Google Flu Trends. <https://en.wikipedia.org/wiki/Google_Flu_Trends>

19. Wilson N, Mason K, Tobias M, Peacey M, Huang Q, Baker M. Interpreting “Google Flu Trends” data for pandemic H1N1 influenza: the New Zealand experience. *Eurosurveillance*. 2009;14(44)

20. Flu search activity (standard deviation from baseline). Updated 2015. <https://www.google.com/publicdata/explore?ds=z3bsqef7ki44ac_&ctype=l&strail=false&nselm=h&met_y=flu_index&scale_y=lin&ind_y=false&rdim=country&idim=country:NL:CL:DE:NZ&tstart=1193529600000&tunit=D&tlen=1106&hl=en&dl=en&iconSize=0.5&uniSize=0.035>

21. National Minimum Dataset (Hospital Events) data dictionary. Wellington: Ministry of Health. <https://www.health.govt.nz/publication/national-minimum-dataset-hospital-events-data-dictionary>

22. Mortality and Demographic Data - series. Wellington: Ministry of Health. <https://www.health.govt.nz/nz-health-statistics/health-statistics-and-data-sets/mortality-and-demographic-data-series>

23. Huang QS, Lopez LD, McCallum L, Adlam B. Influenza surveillance and immunisation in New Zealand, 1997-2006. Article. *Influenza Other Respir Viruses*. Jul 2008;2(4):139-145. doi:10.1111/j.1750-2659.2008.00050.x
