# Supplementary material for: A Framework for Evaluating the Use of Surveillance Systems for Short‐Term Influenza Forecasting
Source: Influenza Other Respir Viruses. 2025 Jul 29;19(8):e70144. doi: 10.1111/irv.70144 (PMC12307093; doi:10.1111/irv.70144)
Supplement: Supplementary file 2 — Appendix S2. Summary of Surveillance System Features, Their Definitions, and Supporting References. [file IRV-19-e70144-s002.docx]

# Appendix 2. Summary of Surveillance System Features, Their Definitions, and Supporting References.

|  | **Category** | **Attribute** | **Definition** | **References** |
| --- | --- | --- | --- | --- |
| **Data quality i.e., features of the data collected by the surveillance system in relation to what it is aiming to measure.** | | | | |
| **1** | **Performance ^1^**  **Effectiveness ^2,3^**  **Technical Implementation ^4^** | **Timeliness** | Timeliness in a public health surveillance system reflects the speed between specified steps, ensuring data is current and readily available ^1,5^. It is defined by the interval between any two steps, which varies based on the purpose of the surveillance ^2,3^. Timeliness can indicate whether the system detects changes swiftly enough to implement risk mitigation measures ^4^. Effective surveillance ensures timely outbreak detection and provides prompt feedback to all relevant stakeholders ^6^. | ^1-6^ |
| **2** | **Performance ^1^**  **Effectiveness ^2,3^**  **Outputs ^4^** | **Sensitivity** | The sensitivity of a surveillance system can be assessed at three levels. First, case detection probability refers to the proportion of actual cases of a disease or health-related event identified by the system within the population. Second, it encompasses the system's capability to identify significant increases in disease occurrence and detect outbreaks, including monitoring changes in case numbers over time. Third, presence detection evaluates the likelihood of detecting a disease if it exists at a specific prevalence level in the population. Overall, sensitivity involves correctly classifying true events ^2-5^. | ^1-6^ |
| **3** | **Effectiveness ^2,3^**  **Outputs ^4^** | **Specificity / False Alarm Rate (inverse of specificity)** | The specificity of a surveillance system refers to its ability to correctly identify true negative cases, meaning it accurately identifies instances where a disease or health-related event is absent ^4^. Specificity assessment involves evaluating the false alarm rate, which is the proportion of non-outbreak periods incorrectly classified as outbreaks ^2,3^. This rate is the inverse of specificity and provides an easily understood measure of the system's accuracy in distinguishing between actual and non-events ^2,3^. Ensuring high specificity is crucial to minimize false alarms, thereby maintaining the reliability and credibility of the surveillance system ^4^. | ^2-4,6,7^ |
| **4** | **Performance ^1^**  **Effectiveness ^2,3^**  **Outputs ^4^** | **Representativeness** | Representativeness ensures that the surveillance data correctly reflects the features of the population of interest ^4,5^. A representative public health surveillance system accurately captures the occurrence of a health-related event over time and its distribution within the population by place and person ^5^. This involves assessing the extent to which the data encompass key characteristics such as population size, production type, age, sex, geographical location, and time of sampling ^2,3^. Evaluating representativeness requires comparing the sample and target populations or specific areas of interest regarding these features and ensuring there is sufficient and accurate data for both ^4^. This aspect is critical for maintaining coverage and minimizing bias in the surveillance system ^4^. | ^1,2,4-6^ |
| **5** | **Effectiveness ^2,3^**  **Outputs ^4^** | **Predictive Value Positive (PVP)** | Predictive Value Positive (PVP) is the proportion of reported cases that truly have the health-related event under surveillance ^5^. PVP is influenced by the sensitivity and specificity of the case definition, which includes the screening and diagnostic tests, and the prevalence of the event in the population ^2-5^. A higher specificity of the case definition typically improves the PVP ^5^. Essentially, PVP represents the likelihood that a detected event is genuine, reflecting the risk of false-positive outcomes in the surveillance system ^4^. An ideal PVP value is 1, indicating perfect accuracy, and is determined by the test characteristics and disease prevalence ^4^. | ^2-5^ |
| **6** | **Effectiveness ^2,3^**  **Outputs ^4^** | **Negative Predictive Value (NPV)** | A negative Predictive Value (NPV) is the probability that no health event is present when no health event is detected by the surveillance system ^2,3^. This value indicates the likelihood of missing the presence of a disease or risk organism, thus reflecting the risk of false-negative outcomes ^4^. The NPV can be influenced by various factors, including the methods of surveillance and the density and geographic spread of the risk organism ^4^. In essence, a high NPV signifies a low probability of undetected cases, which is critical for reliable public health monitoring ^4^. | ^2-4^ |
| **7** | **Performance ^1^** | **Validity** | Validity ensures that the data reflects the true health status and risk factors. Have been discussed and mentioned as a component of the data quality attribute ^5^. | ^5^ |
| **8** | **Performance ^1^**  **Technical Implementation ^4^** | **Completeness** | Completeness refers to the extent to which all required data elements are present and accurately recorded, encompassing geographical, temporal, and demographic dimensions ^5^. In evaluating a surveillance system, data completeness and validity are critical attributes ^5^. Surveillance systems typically collect more than just case counts, incorporating demographic information, details about health-related events, and potential risk factors ^4^. The quality of such data is assessed based on its completeness and validity ^4,5^. Inadequate data completeness may signal issues in data collection, management, or internal communication, potentially impacting the overall effectiveness of the surveillance system ^4^. | ^1,4,5^ |
| **9** | **Effectiveness ^2,3^**  **Technical Implementation ^4^** | **Robustness** | Robustness in a surveillance system refers to its capacity to consistently produce reliable outcomes despite varying levels of uncertainty and assumptions ^5^. This attribute ensures that the system delivers acceptable results across different conditions and over time ^4^. Assessing robustness often involves using info-gap models to evaluate how well the system performs under diverse scenarios of uncertainty ^2,3^. Robustness encompasses high uptime, consistency, and dependability of data, ensuring that the surveillance system remains effective and reliable throughout its operation ^2-4^. | ^2-5^ |
| **10** | **Effectiveness ^2,3^**  **Functional ^2,3^**  **Technical Implementation ^4^** | **Stability / Reliability** | Stability in a public health surveillance system denotes the system's ability to consistently collect, manage, and deliver data accurately without experiencing operational failures ^5^. It refers to reliability which means the system's performance and its capacity to function without interruption ^5^. Stability can be assessed by monitoring the frequency of minor and major faults over a specified period, providing insights into the system’s dependability and uninterrupted functionality ^4^. | ^4,5^ |
| **11** | **Functional ^2,3^**  **Technical Implementation ^4^** | **Availability / Sustainability** | Availability in a public health surveillance system refers to its operational status when required, ensuring that the system is functional and accessible as needed ^2-4^. Availability is typically measured by the proportion of time the system remains fully functional, reflecting its reliability and capacity to support ongoing public health needs ^4^. Sustainability extends beyond immediate availability to encompass the system’s long-term viability, assessing its robustness and enduring functionality over time ^4^. | ^2-4^ |
| **12** | **Effectiveness ^2,3^**  **Design/Structural ^1^**  **Technical Implementation ^4^** | **Coverage** | The proportion of the population of interest (target population) or proportion of areas of interest (e.g. specific habitats or high-risk sites) that is included in the surveillance activity ^2-4^.  The coverage of a surveillance system is related to representativeness, bias, and sensitivity. Coverage can be particularly important in surveillance for the early detection of exotic or new (emerging) diseases or risk organisms ^4^.  A surveillance system is established but does not cover all of the necessary populations or settings ^1^. | ^1-4^ |
| **13** | **Processes ^4^**  **Outputs ^4^** | **Data Quality** | Data quality in a public health surveillance system pertains to the completeness and validity of the recorded data ^5^. This attribute is crucial for ensuring accurate and reliable information, which is fundamental for effective decision-making and response ^4,5^. Data quality is closely linked to data management and storage practices and is integral to the broader RARR attributes—Reliability, Availability, Repeatability, and Robustness—each of which contributes to the overall efficacy and dependability of the surveillance system ^4^. | ^4-6^ |
| **14** | **Functional ^2,3^**  **Technical Implementation ^4^** | **Compatibility / Interoperability** | Compatibility refers to a surveillance system's ability to integrate data seamlessly from various sources and components ^4^. This involves ensuring that the system can effectively merge records from different databases and information systems, utilizing standard technical requirements for interoperability ^4^. Essential elements such as record keys, including sample IDs, NHIs, and postal codes, are crucial for accurate data integration and merging, facilitating comprehensive and cohesive data analysis ^4^. | ^2-4^ |
| **15** | **Effectiveness ^2,3^**  **Outputs ^4^** | **Accuracy / Bias** | Errors and bias can impact a surveillance system at various stages, including case ascertainment and reporting practices, which may vary by time, location, or healthcare provider ^5^. Such biases can distort prevalence estimates and lead to misleading conclusions about the health-related events under surveillance ^2,3^. According to CDC guidelines, these issues fall under the representativeness attribute ^5^. Bias, which can manifest as either information or selection bias, affects how closely a prevalence estimate aligns with the true prevalence, with increased representativeness helping to reduce bias ^4^. The precision of a numerical estimate is influenced by factors such as sample size, confidence level, and data completeness, and any bias can result in inaccurate assessments of disease burden and distribution ^4^. | ^2-5,7,8^ |
| **16** | **Outputs ^4^** | **Historical Data** | The quality and accessibility of archived data are critical for effective research and trend analysis ^4^. This attribute pertains to data management, storage, and the RARR (Reliability, Availability, Repeatability, Robustness) framework ^4^. Key considerations include the number of years data are stored, the completeness and reliability of these data, and whether they are organized in a manner that facilitates necessary interrogation and analysis ^4^. Additionally, a summary overview of the data, along with explanations of key idiosyncrasies and changes in collection methods over time, enhances the utility of historical surveillance data for ongoing and future applications ^4^. | ^4^ |
| **Surveillance system quality, i.e., features related to the wider operation of the surveillance system including its value in relation to the needs of end-users, management, resourcing, and sustained operation.** | | | | |
| **17** | **Performance ^1^** | **Usefulness** | Usefulness refers to the extent to which a public health surveillance system leads to actionable outcomes ^5^. It measures the impact of data analysis and interpretation on public health decisions and actions ^5^. This attribute is closely linked to data management and storage, as the quality and organization of data directly influence the system's ability to produce meaningful and actionable insights ^4^. | ^1,4,5^ |
| **18** | **Functional ^2,3^** | **Simplicity** | The simplicity of a public health surveillance system encompasses both its structural design and operational ease ^5^. An effective surveillance system should be designed to be as straightforward as possible while still fulfilling its intended objectives ^5^. This includes ensuring that the system's structure facilitates smooth data flow and ease of use, thus enhancing its overall functionality and efficiency ^2,3^. | ^2,3,5^ |
| **19** | **Value ^2,3^** | **Cost** | Cost refers to the financial resources and funding required to operate a surveillance system ^5^. It encompasses two primary components: the losses due to the disease, such as mortality, and the resources needed for disease detection, including time, services, and consumables ^2,3^. Economic evaluation compares these resources with disease losses to achieve optimal economic efficiency ^2,3^. Estimating the total economic cost from both losses and expenditures is termed a disease economic impact assessment while estimating only resource expenditures is called a cost analysis ^2,3,5^. | ^2,3,5^ |
| **20** | **Value ^2,3^**  **Impact ^4^** | **Benefit** | Benefit refers to the monetary and non-monetary positive direct and indirect outcomes produced by a surveillance system ^2,3^. This includes financial savings, optimal resource use, and losses avoided due to the system's information ^2-4^. Direct benefits involve improved outbreak response and maintaining a professional network and critical infrastructure for disease control ^4^. Indirect benefits include an enhanced understanding of diseases or risk organisms ^4^. Evaluating benefits also involves assessing whether users feel their requirements are met through the system's outputs ^2-4^. | ^2-4^ |
| **21** | **Value ^2,3^**  **Impact ^4^** | **Efficiency** | Efficiency in a surveillance system refers to achieving an optimal balance between economic investments and the system's outputs ^4^. A system is considered efficient when it meets desired quality attributes, such as precision and timeliness, with minimal resource expenditure ^4^. This balance ensures that the system's operations are cost-effective while maintaining high standards of performance and data quality ^4^. | ^2-4^ |
| **22** | **Organizational ^2,3^**  **Leadership and Resource ^1^**  **Organization and Management ^4^**  **Processes ^4^** | **Surveillance System Organization / Leadership and organizational, workforce / Resource Availability and Technical Competency and Training** | Organizational structure and resource allocation in a surveillance system encompass sector leadership, coordination, mandates, and supportive structures, including trained personnel in critical areas like laboratory services and information technology ^1^. An effective surveillance system is managed through clear objectives, steering and technical committees with relevant expertise, and well-defined roles and responsibilities ^2,3^. It ensures stakeholder involvement, efficient data management, and information dissemination. Adequate financial and human resources are essential, with personnel possessing the necessary expertise and access to ongoing training ^4^. Clearly documented responsibilities for resource provision and alignment of available resources with current requirements are crucial for optimal surveillance operations ^4^. | ^1-4,6^ |
| **23** | **Processes ^4^**  **Analysis and Communication ^1^** | **Data Analysis** | The analysis and interpretation of data within a surveillance system should employ appropriate methods, ranging from basic descriptive statistics to more sophisticated statistical approaches such as time series and spatial analyses ^4^. Effective surveillance systems not only collect data but also analyze and disseminate information in a manner that supports informed decision-making and effective action ^1^. The level of analytical rigor is crucial for understanding trends and patterns, thereby enhancing the system's overall effectiveness and utility ^1,4^. | ^1,4-6^ |
| **24** | **Organizational ^2,3^**  **Processes ^4^** | **Data Management** | Data management encompasses the processes and systems used for data collection, storage, and analysis ^4^. It involves the appropriate use and documentation of protocols for data processing, verification, storage, and backup ^4^. Effective data management ensures the quality, accessibility, usefulness, and security of data ^4^. This attribute requires a thorough understanding of the systems employed to manage and maintain data, highlighting the importance of robust procedures for data collation, storage, and maintenance ^4^. | ^2-6^ |
| **25** | **Processes ^4^** | **Laboratory Management / Field and Laboratory Services** | Laboratory management in surveillance involves coordinating and overseeing laboratory activities to ensure quality assurance and the timely and accurate production of results ^4^. This includes the implementation of appropriate methods for field and laboratory tasks, and adhering to standards that guarantee reliable and prompt outcomes ^4^. Effective management is crucial for maintaining the integrity and efficiency of laboratory operations within the surveillance system ^4^. | ^4,6^ |
| **26** | **Impact ^4^** | **Internal**  **Communication** | Assessment of data and information dissemination within a surveillance system involves evaluating how effectively relevant stakeholders receive and utilize the data and information provided ^4^. The goal is to ascertain that stakeholders have access to accurate, timely, and clarified data and information to perform their roles effectively within the surveillance framework ^4^. | ^4^ |
| **27** | **Analysis and Communication ^1^**  **Impact ^4^** | **External**  **Communication**  **and**  **Dissemination** | Assessment of data and information dissemination to external stakeholders involves evaluating how effectively and promptly the surveillance system communicates relevant data and findings beyond its internal network ^4^. This includes reviewing the types and timeliness of outputs produced, as well as the methods used for their dissemination, such as web-based platforms ^4^. The evaluation aims to ensure that external stakeholders receive accurate and timely information necessary for informed decision-making and public health interventions ^4^. | ^1,4,6^ |
| **28** | **Functional ^2,3^**  **Technical Implementation ^4^** | **Acceptability / Engagement** | Acceptability in a public health surveillance system pertains to the extent of willingness among individuals and organizations to engage with the system ^5^. It involves evaluating how stakeholders are involved in the system’s planning, design, and implementation, and assessing their perceptions regarding the benefits or drawbacks of participation ^4^. This attribute also considers whether stakeholders are adequately informed about the system and its functions ^4^. Additionally, it includes examining factors that may influence participation levels, such as compensation for the consequences of disease detection and the overall impact of these factors on stakeholder engagement ^2,3^. | ^1-6^ |
| **29** | **Functional ^2,3^**  **Organization and Management ^4^** | **Flexibility / Adaptability** | Flexibility in a public health surveillance system refers to its capacity to adjust to evolving information needs and operational conditions with minimal additional resources, such as time, personnel, or funds ^4^. This attribute encompasses the system's ability to integrate new health hazards, accommodate changes in case definitions or technology, and adapt to shifts in funding sources or reporting methods ^2,3,5^. Systems with greater flexibility can more readily handle these changes and maintain effectiveness without requiring substantial modifications or investments ^2,3,5^. Generally, simpler or more generic systems are better positioned to adapt to varying circumstances ^4^. | ^2-5^ |
| **30** | **Socio-Ethical ^7^** | **Transparency** | Transparency in evaluating public health surveillance systems refers to the clarity and openness with which the processes, methods, and findings of the system are communicated ^9^. This attribute encompasses the extent to which stakeholders can access and understand information about data collection, analysis, and decision-making procedures. Transparency ensures that the surveillance system's operations are visible and understandable, fostering trust and accountability among users and the public. It involves clear documentation and reporting of methodologies, data sources, and outcomes, as well as openness about limitations and uncertainties in the data and analysis. | ^7,9^ |
| **31** | **Socio-Ethical ^7^** | **Accountability** | Accountability in evaluating public health surveillance systems refers to the obligation of individuals and organizations involved in the surveillance process to be answerable for their actions and decisions. This attribute involves establishing clear responsibilities and expectations for all parties, ensuring that they are held responsible for the accuracy, reliability, and ethical management of data. Accountability includes mechanisms for oversight and review, such as audits and evaluations, to verify that the system meets its objectives and adheres to established standards. It also entails transparent reporting of system performance and outcomes, enabling stakeholders to assess the effectiveness and integrity of the surveillance activities. | ^7-9^ |

**References**

1. Baker MG, Easther S, Wilson N. A surveillance sector review applied to infectious diseases at a country level. *BMC Public Health*. 2010/06/11 2010;10(1):332. doi:10.1186/1471-2458-10-332

2. Peyre M, Hoinville L, Njoroge J, et al. The RISKSUR EVA tool (Survtool): A tool for the integrated evaluation of animal health surveillance systems. *Preventive Veterinary Medicine*. 2019/12/01/ 2019;173:104777. doi:<https://doi.org/10.1016/j.prevetmed.2019.104777>

3. Peyre M, Salman M, Steneroden K. Frameworks and tools for evaluating health surveillance systems. *Principles for Evaluation of One Health Surveillance: The EVA Book*. Springer; 2022:43-60.

4. Muellner P, Stärk KD, Watts J. *Surveillance Evaluation Framework (SurF): Main Document*. Ministry for Primary Industries; 2016.

5. German RR, Lee LM, Horan JM, Milstein RL, Pertowski CA, Waller MN. Updated guidelines for evaluating public health surveillance systems: recommendations from the Guidelines Working Group. *MMWR Recomm Rep*. Jul 27 2001;50(Rr-13):1-35; quiz CE1-7.

6. World Health O. *Instructions for the national infection prevention and control assessment tool 2 (IPCAT2)*. 2017. 2017. <https://iris.who.int/handle/10665/330078>

7. Muley A, Muzumdar P, Kurian G, Basyal GP. Risk of AI in Healthcare: A comprehensive literature review and study framework. *arXiv preprint arXiv:230914530*. 2023;

8. Pearson A. How to use AI and personal data appropriately and lawfully. 2022;

9. Cox JM, F. Machine Learning & Big Data Laws and Regulations 2024. *Global Legal Insights*. 2024;
