# Supplementary material for: A Framework for Evaluating the Use of Surveillance Systems for Short‐Term Influenza Forecasting
Source: Influenza Other Respir Viruses. 2025 Jul 29;19(8):e70144. doi: 10.1111/irv.70144 (PMC12307093; doi:10.1111/irv.70144)
Supplement: Supplementary file 3 — Appendix S3. Attribute Definitions and Ranking Criteria for Evaluating Surveillance System Suitability for Training and Short‐term Forecasting. [file IRV-19-e70144-s001.docx]

# Appendix 3. Attribute Definitions and Ranking Criteria for Evaluating Surveillance System Suitability for Training and Short-term Forecasting

A thorough review of established evaluation frameworks from organizations such as the World Health Organization (WHO), the Centers for Disease Control and Prevention (CDC), and other national and global published literature was carried out ^1-9^. These frameworks are valuable for assessing how well surveillance systems meet their public health objectives. However, they do not specifically address the suitability of a system's data for machine learning (ML) or short-term forecasting. To address this, we developed a complementary approach focused on the attributes most relevant to AI/ML applications.

From these existing frameworks, we identified a total of 31 unique attributes. In the context of AI/ML, the primary task is to collect, clean, and prepare data for model input, making data quality an important characteristic. Poor-quality data can reduce model performance and result in unreliable predictions. Based on this, we first filtered the 31 attributes to retain the 16 most directly related to data quality (see Appendix 2). These were further refined to a final list of eight key attributes considered important for both AI/ML applications: timeliness, sensitivity, specificity, representativeness, coverage, robustness, completeness, and historical data. Three criteria guided the selection of these eight attributes: (1) Their repeated appearance across multiple surveillance evaluation frameworks; (2) Their alignment with the specific data needs of AI/ML models; (3) Expert judgment from a multidisciplinary panel, including epidemiologists, public health experts, virologist, and data scientists who have years of experience working with these systems.

To evaluate each surveillance system, raw scores were assigned through a comprehensive review of publicly available documentation, including surveillance protocols, government reports, and peer-reviewed literature. The scoring process involved internal discussions among the multidisciplinary research team. Through a series of iterative discussions, we reached consensus on the raw scores for each attribute and documented the reasoning behind them. Through this process we ensured the scoring was consistent and transparent (see Appendix 1 and Table 1 in the main text for the reasoning behind the raw scores for each surveillance system).

Applying machine learning algorithms for short-term forecasting requires two datasets. The first is used to train the machine learning model. The second is used as input for the trained model to compute the forecast. The datasets are structurally the same, but the two tasks prioritize different qualities, which motivated the use of separate sets of evaluation metrics:

- Model training benefits from large volumes of accurate historical data. Attributes such as length of historical data, sensitivity, specificity, and completeness are especially important here to ensure the model can learn meaningful patterns from the past.
- Short-term forecasting, however, depends on timely and stable data. In this context, timeliness becomes the highest priority, along with robustness, sensitivity, and specificity, to ensure the data feeding into the model is current and accurate.

For each metric, we assigned predefined multipliers to the eight attributes to reflect their relative importance. Assuming equal importance for all the attributes, we would assign a weight of 0.125 (1 divided by 8). However, not all attributes are equally important for every task, so we defined priority bands: Low priority: $<0.125$; intermediate priority: $\geq0.125 and<0.25$; critical priority: $\geq0.25$. This approach ensures that the most important attributes for each application have a bigger impact on the final score.

To calculate overall scores, we applied these multipliers to the raw scores assigned for each attribute of the surveillance systems. The weighted scores were then summed, ensuring the total remained consistent with the five-level scoring system. This allowed us to compare how well different surveillance systems support model training and short-term forecasting in a standardized, interpretable way.

**Table 1. Attribute Definitions and Ranking Criteria**

|  | **Attribute** | **Definition** | **Ranking Definition (Score)** |
| --- | --- | --- | --- |
| **1** | **Timeliness** | Timeliness in a public health surveillance system refers to the speed at which data is collected, processed, and made available, ensuring that the information is current and actionable ^1,4^. It is defined by the interval between any two key steps in the surveillance process, which can vary depending on the system’s specific purpose ^2,3^. Timeliness is important for determining whether the system can detect changes quickly enough to allow for the implementation of effective risk mitigation measures ^5^. An effective surveillance system ensures that outbreaks are detected promptly and that timely feedback is provided to all relevant stakeholders, facilitating rapid response ^5,9^. | - **High (5):** The system consistently provides data with minimal delay (real-time). - **Moderately high (4):** The system generally delivers timely data, though occasional delays may occur (less than a fortnight). - **Moderate (3):** The system provides data in a reasonable timeframe, though delays are common (more than a fortnight and less than a month). - **Moderately low (2):** The system often experiences delays, significantly hindering prompt detection (more than a month and less than three months). - **Low (1):** The system regularly provides data with considerable delay, greatly compromising its utility in timely public health interventions (more than three months). |
| **2** | **Sensitivity** | Sensitivity in a surveillance system is evaluated at three levels. First, case detection probability measures the proportion of actual cases of a disease or health-related event that the system successfully identifies within the population. Second, outbreak detection capability assesses the system's ability to recognize significant increases in disease occurrence and detect outbreaks, including its effectiveness in monitoring temporal changes in case numbers. Third, presence detection evaluates the system's likelihood of identifying a disease if it exists at a specific prevalence level within the population. Overall, sensitivity reflects the system's effectiveness in correctly identifying true events and minimizing false negatives ^2-5^. | - **High (5):** The system consistently identifies the majority of true cases with minimal false negatives. - **Moderately high (4):** The system generally identifies most true cases though some minor false negatives may occur. - **Moderate (3):** The system identifies a reasonable proportion of true cases with some noticeable false negatives. - **Moderately low (2):** The system frequently misses true resulting in significant false negatives. - **Low (1):** The system often fails to identify true with a high rate of false negatives. |
| **3** | **Specificity / False Alarm Rate (inverse of specificity)** | Specificity in a surveillance system refers to its ability to accurately identify true negative cases, meaning it correctly identifies instances where a disease or health-related event is absent ^5^. This attribute is assessed by evaluating the false alarm rate, which represents the proportion of non-outbreak periods that are incorrectly classified as outbreaks ^2,3^. The false alarm rate is the inverse of specificity and provides a straightforward measure of the system's accuracy in distinguishing between actual events and non-events ^2,3^. High specificity is essential for minimizing false alarms, thereby enhancing the reliability and credibility of the surveillance system ^5^. | - **High (5):** The system consistently identifies non-outbreak periods with high accuracy, resulting in a very low false alarm rate. - **Moderately high (4):** The system generally identifies non-outbreak periods accurately, though occasional false alarms may occur. This reflects a good overall accuracy in distinguishing between outbreaks and non-events. - **Moderate (3):** The system shows reasonable performance in identifying non-outbreak periods, but with a noticeable false alarm rate, which affects its accuracy in distinguishing between actual and non-events. - **Moderately low (2):** The system frequently misclassifies non-outbreak periods as outbreaks, leading to a higher false alarm rate and reduced accuracy in recognizing the absence of disease or events. - **Low (1):** The system often fails to accurately identify non-outbreak periods, resulting in a high false alarm rate. This significantly impacts its ability to correctly distinguish between true and false events. |
| **4** | **Representativeness** | Representativeness in a surveillance system refers to the extent to which the collected data accurately reflects the characteristics of the population of interest ^4,5^. A representative public health surveillance system captures the occurrence of a health-related event over time and its distribution across the population by key factors such as place, person, and time ^4^. This involves evaluating the data to ensure it includes essential characteristics like population size, demographic factors (e.g., age, sex), geographical location, and the timing of data collection ^2,3^. Evaluating representativeness requires a comparison between the sample population and the target population to verify that the data accurately and comprehensively covers the population or areas of interest ^5^. High representativeness is crucial for maintaining broad coverage and minimizing bias, thereby enhancing the reliability of the surveillance system ^5^. | - **High (5):** The system consistently captures data that accurately represents the population of interest across all key characteristics, with minimal bias. - **Moderately high (4):** The system generally captures data that are representative of the population, though some minor biases may exist in certain characteristics. - **Moderate (3):** The system provides a reasonable level of representativeness, but there are noticeable gaps or biases in the data, affecting certain population characteristics. - **Moderately low (2):** The system often fails to adequately represent the population, with significant biases or gaps in key characteristics. - **Low (1):** The system regularly provides data that poorly represents the population, resulting in substantial biases and insufficient coverage of key characteristics. |
| **5** | **Completeness and Linkability** | Completeness in a surveillance system refers to the extent to which all necessary data elements are collected and accurately recorded, encompassing geographical, temporal, and demographic dimensions ^4^. In evaluating a surveillance system, data completeness and validity are critical attributes, as they ensure that the data set is comprehensive and linkable to other systems for enhanced analysis ^4^. Surveillance systems often gather more than just case counts, including demographic details, information about health-related events, and potential risk factors ^5^. The quality of such data is assessed based on its completeness and validity ^4,5^. Incomplete data may indicate issues in data collection, management, or internal communication, potentially impacting the overall effectiveness of the surveillance system ^5^. | - **High (5):** The system consistently records all required data elements with minimal gaps, ensuring comprehensive coverage across all dimensions (geographical, temporal, and demographic) and maintaining linkability to other systems. - **Moderately** **high (4):** The system generally provides complete data, with occasional minor gaps or omissions that do not significantly impact the overall data quality or its ability to be linked to other datasets. - **Moderate (3):** The system provides a reasonable level of data completeness, but there are noticeable gaps in some data elements, affecting the overall quality, usability, and potential for integration with other systems. - **Moderately low (2):** The system often has significant gaps in data collection, leading to incomplete records that hinder effective analysis and limit its linkability to external systems. - **Low (1):** The system regularly fails to collect and record essential data elements, resulting in substantial gaps that compromise the system's reliability, usefulness, and capacity for integration with other datasets. |
| **6** | **Robustness** | Robustness in a surveillance system refers to its capacity to consistently produce reliable outcomes despite varying levels of uncertainty and assumptions ^4^. This attribute ensures that the system delivers acceptable results across different conditions and over time ^5^. Assessing robustness often involves using info-gap models to evaluate how well the system performs under diverse scenarios of uncertainty ^2,3^. Robustness encompasses high uptime, consistency, and dependability of data, ensuring that the surveillance system remains effective and reliable throughout its operation ^2,3,5^. | - **High (5):** The system consistently delivers reliable and accurate results under a wide range of conditions, with minimal impact from uncertainty or assumptions. - **Moderately** **high (4):** The system generally performs reliably, though it may show some minor inconsistencies under certain conditions of uncertainty. - **Moderate (3):** The system provides acceptable results under typical conditions but may struggle with reliability when faced with higher levels of uncertainty. - **Moderately low (2):** The system frequently encounters reliability issues under varying conditions, leading to inconsistencies and reduced dependability. - **Low (1):** The system often fails to deliver reliable results, with significant issues in maintaining consistency and dependability under uncertain conditions. |
| **7** | **Coverage** | The proportion of the population of interest (target population) or proportion of areas of interest (e.g. specific habitats or high-risk sites) that is included in the surveillance activity ^2,3,5^.  This attribute is closely related to representativeness, bias, and sensitivity, as it determines how comprehensively the surveillance system captures relevant data across various populations or locations. Adequate coverage is particularly crucial for the early detection of emerging or exotic diseases and other risk factors ^5^. Insufficient coverage can result in gaps that undermine the system’s effectiveness, especially in identifying and responding to new threats ^1^. | - **High (5):** The surveillance system covers nearly all of the target population or areas of interest, ensuring minimal gaps in data collection. - **Moderately** **high (4):** The system covers most of the necessary populations or areas, though some minor gaps may exist. - **Moderate (3):** The system provides reasonable coverage, but there are noticeable gaps that may affect the comprehensiveness of data collection. - **Moderately low (2):** The system often fails to cover significant portions of the population or areas, leading to substantial gaps in surveillance. - **Low (1):** The system covers only a small portion of the target population or areas, resulting in major deficiencies that limit its overall effectiveness. |
| **8** | **Historical Data** | Historical data refers to the quality, accessibility, and usability of archived surveillance data, which are essential for conducting effective research, trend analysis, and predictive modelling ^5^. This attribute involves the management, storage, and organization of data within the RARR (Reliability, Availability, Repeatability, Robustness) framework ^5^. Critical factors include the number of years for which data are available, the completeness and reliability of these records, and whether they are structured in a way that supports easy interrogation and analysis ^5^. Additionally, comprehensive documentation of the data, including key idiosyncrasies and changes in data collection methods over time, enhances the value of historical data for AI and ML applications, particularly in generating long-term forecasts and learning patterns from past trends ^5^. | - **High (5):** The system provides well-organized, reliable, and complete historical data spanning many years, with clear documentation of changes in collection methods. - **Moderately** **high (4):** The system offers generally reliable and accessible historical data, though some minor gaps or inconsistencies may exist. - **Moderate (3):** The system contains a reasonable amount of historical data, but noticeable gaps, inconsistencies, or incomplete records hinder comprehensive analysis. - **Moderately low (2):** The system has limited historical data, with significant gaps and unreliable records, reducing its usefulness for trend analysis. - **Low (1):** The system offers very little or poorly managed historical data, making it difficult to conduct meaningful analyses or apply AI and ML techniques effectively. |
| **9** | **Usefulness for training** | The usefulness for training represents how well each surveillance system can provide data suitable for training machine learning (ML) algorithms. A higher score indicates that the system is more valuable for developing models with training datasets, making it better suited to contribute effectively to AI/ML forecasting efforts in public health contexts.  Each surveillance system is evaluated on a 5-level scale, based on key attributes, by assigning a weighted score to each attribute using the training multipliers. The raw score for each attribute (ranging from 1 to 5) is multiplied by its respective multiplier, and the resulting values are summed to obtain a final score. | - **High (5):** The system provides excellent support for training ML algorithms, offering high-quality data that significantly enhances AI/ML forecasting accuracy. - **Moderately** **high (4):** The system performs well in training ML algorithms, with minor limitations that do not significantly impact its effectiveness. - **Moderate (3):** The system provides adequate support for ML training but has noticeable limitations in data quality that may affect forecasting. - **Moderately low (2):** The system has substantial limitations in data quality which hinder its usefulness for ML training. - **Low (1):** The system lacks alignment with ML training needs, providing insufficient data for effective AI/ML forecasting. |
| **10** | **Usefulness for Short-term Forecasting** | Usefulness for short-term forecasting refers to how effectively a surveillance system can support short-term predictions (typically over days or weeks (1 to 4 weeks) using real-time or near real-time data. Short-term forecasting relies on timely, accurate data to make immediate forecasts about disease trends or outbreaks, making certain attributes such as timeliness, sensitivity, and specificity particularly important.  Each surveillance system is evaluated on a 5-level scale based on key attributes, with weighted scores assigned using short-term forecasting multipliers. The raw score for each attribute (ranging from 1 to 5) is multiplied by its respective multiplier, and the resulting values are summed to obtain a final score for Usefulness for Short-term Forecasting. | - **High (5):** The system provides real-time or near real-time high-quality data, suitable for accurate, short-term forecasting. - **Moderately** **high (4):** The system performs well for short-term forecasting but has some minor delays or data quality issues that impact short-term forecasts. - **Moderate (3):** The system provides some support for short-term forecasting, but limitations in timeliness or data quality may affect the reliability of forecasts. - **Moderately low (2):** The system has significant delays or data limitations, making it less effective for short-term forecasting. - **Low (1):** The system is not well-suited for short-term forecasting, with major issues in timeliness, accuracy, or data availability. |

**Table 2. Multipliers and rationales for weighting surveillance data metrics for assessing the usefulness of surveillance data sources for Training and Short-term Forecasting**

| **Attributes** | **Usefulness for Training Metric** | | | **Usefulness for Short-term Forecasting** | | |
| --- | --- | --- | --- | --- | --- | --- |
|  | **Multiplier** | **Priority Band** | **Rationale** | **Multiplier** | **Priority Band** | **Rationale** |
| **Timeliness** | 0.05 | Low | In model development, data can be processed routinely, allowing for time lags without impacting model training. | 0.25 | Critical | Real-time or near real-time data availability is essential for short-term forecasting. |
| **Robustness** | 0.10 | Low | While beneficial, robustness is of low priority during the training process as models can be retrained with updated data if inconsistencies arise. | 0.15 | Intermediate | Consistent and reliable data outcomes are important in real-time applications to ensure dependable forecasts. |
| **Sensitivity** | 0.15 | Intermediate | Prioritizing sensitivity ensures that the model learns from true positive cases, improving its ability to detect actual events more accurately. | 0.15 | Intermediate | Prioritizing sensitivity reduces the risk of missed events in forecasts. |
| **Specificity** | 0.17 | Intermediate | Prioritizing specificity, minimizes false positives during training, and prevents the model from learning incorrect patterns. | 0.15 | Intermediate | Prioritizing sensitivity reduces false alarms in forecasts. |
| **Historical Data** | 0.18 | Intermediate | Historical data provide a rich dataset for the model to learn patterns, making it a high priority for the training process. | 0.05 | Low | For short-term forecasts, current data trends are more influential. |
| **Representativeness** | 0.10 | Low | While beneficial, representativeness is of lower priority during training, as models can adjust for certain biases. | 0.10 | Low | While beneficial, representativeness is less critical, provided the data captures the current trends accurately. |
| **Coverage** | 0.10 | Low | Sufficient coverage enhances model generalizability, but its impact is less critical during the training phase. | 0.05 | Low | For short-term forecasts, even partial coverage can be sufficient if the data is timely and relevant. |
| **Completeness** | 0.15 | Intermediate | Completeness ensures the model has access to all relevant data fields, enhancing its ability to learn patterns effectively. | 0.10 | Low | While beneficial, some missing data can be tolerated in short-term forecasts, without significantly impacting accuracy. |

**References**

1. Baker MG, Easther S, Wilson N. A surveillance sector review applied to infectious diseases at a country level. *BMC Public Health*. 2010/06/11 2010;10(1):332. doi:10.1186/1471-2458-10-332

2. Peyre M, Hoinville L, Njoroge J, et al. The RISKSUR EVA tool (Survtool): A tool for the integrated evaluation of animal health surveillance systems. *Preventive Veterinary Medicine*. 2019/12/01/ 2019;173:104777. doi:<https://doi.org/10.1016/j.prevetmed.2019.104777>

3. Peyre M, Salman M, Steneroden K. Frameworks and tools for evaluating health surveillance systems. *Principles for Evaluation of One Health Surveillance: The EVA Book*. Springer; 2022:43-60.

4. German RR, Lee LM, Horan JM, Milstein RL, Pertowski CA, Waller MN. Updated guidelines for evaluating public health surveillance systems: recommendations from the Guidelines Working Group. *MMWR Recomm Rep*. Jul 27 2001;50(Rr-13):1-35; quiz CE1-7.

5. Muellner P, Stärk KD, Watts J. *Surveillance Evaluation Framework (SurF): Main Document*. Ministry for Primary Industries; 2016.

6. Cox JM, F. Machine Learning & Big Data Laws and Regulations 2024. *Global Legal Insights*. 2024;

7. Muley A, Muzumdar P, Kurian G, Basyal GP. Risk of AI in Healthcare: A comprehensive literature review and study framework. *arXiv preprint arXiv:230914530*. 2023;

8. Pearson A. How to use AI and personal data appropriately and lawfully. 2022;

9. World Health O. *Instructions for the national infection prevention and control assessment tool 2 (IPCAT2)*. 2017. 2017. <https://iris.who.int/handle/10665/330078>
